# Direct evaluation of the electrocardiographic spatial QRS-T angle without the need for orthogonal transformation

**DOI:** 10.1038/s41598-026-37361-w

**Published:** 2026-02-04

**Authors:** Jan Řehoř, Katerina Hnatkova, David Pospíšil, Kateřina Helánová, Peter Smetana, Georg Schmidt, Irena Andršová, Marek Malik

**Affiliations:** 1 Department of Internal Medicine and Cardiology, University Hospital Brno, Jihlavská 20, 625 00 Brno, Czech Republic; 2 Department of Internal Medicine and Cardiology, Faculty of Medicine, Masaryk University, Jihlavská 20, 625 00 Brno, Czech Republic; 3 National Heart and Lung Institute, Imperial College London, 72 Du Cane Rd, Shepherd’s Bush, London, W12 0NN England; 4 Wilhelminenspital der Stadt Wien, Montleartstraße 37, 1160 Vienna, Austria; 5 Klinikum rechts der Isar, Technische Universität München, Ismaninger Straße 22, 81675 Munich, Germany

**Keywords:** Electrocardiogram, QRS-T angle, Orthogonal transformation, Direct computation, Stability, Reproducibility, Computational biology and bioinformatics, Physiology, Cardiology

## Abstract

Increased electrocardiogram (ECG) spatial QRS-T wave angle is a recognised risk factor. Standard evaluation of the angle requires deriving orthogonal ECG leads, either by general transformation matrices into XYZ leads or by singular value decomposition (SVD). This study shows that the transformation is not needed, and that the spatial QRS-T angle can be calculated directly from the original ECG leads. The direct computation was tested using long-term 12-lead ECGs of 523 healthy volunteers (259 females). A total of 659,313 individual 10-second ECG samples were obtained providing 7,350,733 individual beats which were analysed both by the direct method using 8 algebraically independent leads and by the conventional XYZ and SVD transformations. On average, the results of the direct non-transformation method were closer to the SVD-based results (averaged differences below 1 degree) than to the XYZ-based results (averaged differences below 2 degrees). The subject-specific regressions to the underlying heart rate showed that the proposed direct method was significantly more reproducible (*p* < 0.0001) and that it showed more compact variability within individual ECG samples (*p* < 0.0001). Thus, the study shows not only that the QRS-T angle can be computed without any orthogonal transformation but that the results of the direct computation are also more precise.

## Introduction

The angle between the spatial orientations of the QRS complex and of the T wave was initially introduced, as a part of the so-called ventricular gradient, by Willson et al. already in 1934^1^. Although the concept was subsequently formally refined by Geselowitz et al. in 1983^2^, its physiologic and mainly clinical value had long been considered inconsequential, despite repeated suggestions that the angle represents a global measure of ventricular action potential variations^3^ and that it might indicate vulnerability to arrhythmia^4^. The clinical risk-prediction value of the spatial QRS-T angle was published in 2000 when Zabel et al. reported that in survivors of acute myocardial infarction, increased QRS-T angle indicated significant mortality risk independently of other risk factors^5^. Since then, the risk predictive value of the QRS-T angle increases has been shown in a broad spectrum of clinical conditions including general population^6^, patients with coronary artery disease^7^, stable angina^8^, systemic sclerosis^9^, hypertrophic cardiomyopathy^10^, heart failure^11^, as well as haemodialyzed patients^12,13^, and in a number of other conditions.

Some studies simplified the spatial QRS-T angle and used only calculation of standard ECG vectors in the frontal plane^14^. Nevertheless, all the studies that we have just mentioned used the true spatial QRS-T angle^5–13^. To derive the spatial angle from a standard 12-lead ECG, transformation of the multi-lead signal into 3 orthogonal directions has always been used. Two types of such a transformation have been employed. General transformation utilizes conversion matrices^15–18^ that derive orthogonal anatomically defined XYZ leads, i.e., leads corresponding to the Frank lead system^19^. Signal-specific transformation is based on singular value decomposition (SVD) that converts the algebraically independent leads of the 12-lead ECG (i.e., leads I, II, V1, …, V6) into algebraically orthogonal leads S1, …, S8^20,21^, allowing to consider the dominant derived leads S1, S2, and S3 as a rotation of the anatomic leads XYZ to fit the major spatial components of the original multi-lead ECG signal. Three orthogonal leads subsequently allow the calculation of the QRS-T angle between the spatial loops of the QRS complex and of the T wave (see details in the Methods section).

The transformation of the source 12-lead signal into a derived system of 3 orthogonal leads is a recognised methodological complexity. Indeed, suggestions have been proposed of how to approximate the spatial QRS-T angle from the native 12-lead ECGs^22,23^. Having this in mind, this study suggests that no ECG transformation is needed to obtain the value of the spatial QRS-T angle and that the method of computing the angle directly from the original multi-lead ECG signal also increases the precision of the QRS-T angle values.

Recently, it has been shown that not only QRS-T angle width but also its beat-to-beat variability provides prognostic information^24^. We have therefore investigated also the beat-to-beat variability of the angle in 10-second ECGs, realising that such short-term recordings are the most frequent ECG mode used in clinical practice^25^.

A previous investigation by our team studied the stability of different expressions of the QRS-T angle based on general and on signal-specific orthogonal 3-dimensional ECG representations^26^. The present study used the same dataset. In the previous study, we used intra-subject regression analyses of the QRS-T angles to the underlying heart rate (expressed by RR interval durations) and proposed that the statistical evaluation of the regression residua is a meaningful way of comparing the precision of the different possible expressions of the QRS-T angle^26^. Here, we employ the same analytical scheme to compare the precision of the newly proposed method of direct QRS-T angle calculation with the previously investigated computation methods based on the conversion to 3 orthogonal leads.

## Methods

### Study population

As reported^26^, clinical pharmacology studies conducted at 3 different research sites enrolled 523 healthy volunteers including 259 females. Before enrolment, participants had a normal clinical ECG and normal clinical investigation. All the source studies used mutually consistent protocols in respect of drug-free baseline days and employed the same standard inclusion and exclusion criteria of Phase I pharmacology investigations^27^.

The source studies fully satisfied the Helsinki declaration. Their protocols, conduct, clinical procedures, experimental parts of the protocols, and the adherence to the Helsinki declaration were approved by institutional ethics and licensing bodies (Parexel in Baltimore; California Clinical Trials in Glendale; and Spaulding in Milwaukee—approval letters were issued to the study sponsors). Consistent with the Helsinki declaration, all subjects gave informed written consent to study participation including subsequent scientific investigations of collected data. Consistent with the permissions by the sponsors of the original studies, only drug-free data were used in the research reported here making further details of the source studies irrelevant.

### ECG recordings

Repeated long-term 12-lead Holter ECG recordings with Mason-Likar electrode positions were obtained during full day-time periods while the subjects were on no medication and while they were, by protocol, not allowed to smoke and/or consume alcohol or caffeinated drinks and/or to sleep. The study protocols were mutually consistent in respect of the conduct during the drug-free days.

Using previously tested procedures^28,29^, multiple non-overlapping 10-s ECG segments were extracted from the long-term ECGs. All extracted segments contained only ectopic-free sinus rhythm and were obtained from (a) pre-specified time-points of the pharmacologic studies, and (b) recording scans aimed at finding different underlying heart rates. Distinction between those segments that were and were not preceded by heart rate changes exceeding ± 2 beats per minute (bpm) over a 5-min period were made. The extracted ECG segments were used during further data processing only if satisfactory algorithmic measurement of QRS complexes and QT intervals was possible^28,29^.

### QRS and T wave measurements

In each of the extracted ECG segments, QRS complex and the T wave offset were identified by validated procedures^28,29^ that included repeated visual controls, manual corrections, and intra- and inter-subject repeatability and stability of the measurements. Consistency of the ECG morphology interpretation was also confirmed^30^. Visual verifications of the measurements were made in the representative median waveforms of the 10-s segments (sampled at 1000 Hz) with the superimposition of all 12 leads on the same isoelectric axis^31,32^.

Using previously proposed techniques^33,34^, interval delineations (i.e., QRS onset and offset, and T wave offset) were projected to individual beats within the selected 10-s ECG segments requiring the maximum correlation between the median waveform and the signal of individual QRS-T complexes. It was previously reported that this projection process might be lead dependent^35^. Therefore, the cross-correlation technique was applied to the vector magnitude of orthogonal leads derived by general conversion applicable to Mason-Likar ECG configuration^18^.

### Concepts of QRS-T angle computation

Similar to simple frontal planar QRS-T angles, the spatial angles are based on elementary concept of angles between vector orientations. As well known, regardless of the dimension of vectors $\:\mathfrak{a}$ and $\:\mathfrak{b}$, their angle $\:\mathfrak{\sphericalangle\:}(\mathfrak{a},\mathfrak{b})$ is given by the formula $\:\sphericalangle\:\left(\mathfrak{a},\mathfrak{b}\right)=\mathrm{a}\mathrm{c}\mathrm{o}\mathrm{s}\left(\frac{\mathfrak{a}\bullet\:\mathfrak{b}}{\left|\mathfrak{a}\right|\left|\mathfrak{b}\right|}\right)$, where $\:\mathfrak{c}\bullet\:\mathfrak{d}$ is the scalar dot product of vectors $\:\mathfrak{c}\mathfrak{\:}$and $\:\mathfrak{d}$, and $\:\left|\mathfrak{e}\right|$ is the vector magnitude of vector $\:\mathfrak{e}$.

There are two principal approaches of computing the QRS-T angles. Either, single vectors $\:\mathfrak{Q}$ and $\:\mathfrak{T}\mathfrak{\:}$(e.g., based on waveform integrals or on maximal vector magnitude) are assigned to the QRS complex and the T wave, respectively, and their angle $\:\mathfrak{\sphericalangle\:}(\mathfrak{Q},\mathfrak{T})$ is calculated, or, a vector $\:\mathfrak{E}\left(t\right)\:$is assigned to each multi-dimensional ECG sample $\:t$ and dual integrative approach is applied to the sequences of these vectors within the QRS complex and T wave^21,26,36^. We have previously proposed the integrative approach in the form

$$\:\left({\int\:}_{t=Q}^{J}{\int\:}_{u=J}^{{T}_{e}}\sphericalangle\:\left(\mathfrak{E}\left(t\right),\mathfrak{E}\left(u\right)\right)\cdot\:\left|\mathfrak{E}\left(t\right)\right|\cdot\:\left|\mathfrak{E}\left(u\right)\right|\:du\:dt\right)/\left({\int\:}_{t=Q}^{J}{\int\:}_{u=J}^{{T}_{e}}\left|\mathfrak{E}\left(t\right)\right|\cdot\:\left|\mathfrak{E}\left(u\right)\right|\:du\:dt\right)$$.

(where $\:Q$, $\:J$, and $\:{T}_{e}$ are the QRS onset, QRS offset, and T wave offset, respectively) and have shown that when combined with both general and subject-specific ECG transformations into 3-dimensional lead systems, it provided results that were more stable than the computations based on single QRS and T wave vectors^26^.

### Lead systems

In this study, we considered 4 different lead configurations for QRS-T complexes of each beat in the selected 10-second ECG segments. Specifically, we considered the following 4 sets of ECG leads:

(A) Using the general transformation matrix suitable for Mason-Likar electrode configuration^18^, orthogonal leads *X*, *Y*, and *Z* (i.e., leads approximating the Frank system of right → left, front → back, and head → foot orientations)^19^ were computed, creating a 3-dimensional lead system $\:{\mathcal{L}}_{\mathrm{X}\mathrm{Y}\mathrm{Z}\:}$= {*X*,*Y*,*Z*}. That is, each of the orthogonal leads *X*, *Y*, and *Z* was obtained as an algebraic combination of the original ECG leads using the published transformation constants^18^.

(B) The algebraically independent leads of the 12-lead ECG (i.e., leads I, II, V1, V2, …, V6) were processed by the SVD transformation^20,21,37,38^ between the QRS onset and T wave offset of the given QRS-T pattern. This derived 3 dominant and mutually orthogonal leads *S1*, *S2*, and *S3* (as if the system of orthogonal axes was optimally rotated for the given 12-lead signal). A 3-dimensional lead system $\:{\mathcal{L}}_{\mathrm{S}\mathrm{V}\mathrm{D}\:}$= {*S1*, *S2*, *S3*} was created in this way.

(C) Instead of using only the dominant orthogonal leads derived by the SVD transformation, all 8 algebraically orthogonal leads were considered and an 8-dimensional lead system $\:{\mathcal{L}}_{\mathrm{S}\mathrm{V}\mathrm{D}-8\:}$= {*S1*, *S2*,…, *S8*} was used.

(D) Finally, we used the original algebraically independent ECG leads without applying any transformation and thus created an 8-dimensional lead system $\:{\mathcal{L}}_{8-\mathrm{l}\mathrm{e}\mathrm{a}\mathrm{d}\mathrm{s}\:}$= {I, II, V1, V2, … V6}.

### QRS-T angle evaluations

For each of lead system $\:{\mathcal{L}}_{\dots\:\:}$of $\:\mathcal{n}$ ECG leads (i.e., either $\:\mathcal{n}=3$ or $\:\mathcal{n}=8$) we considered two vectorial representations for the purposes of QRS-T angle evaluations.

Firstly, we created global QRS and T wave $\:\mathcal{n}$-dimensional vectors based on calculating the areas under the signals of individual leads. That is, formally, we created QRS complex and T wave vectors $\:{\mathfrak{Q}=\left[{\int\:}_{t=Q}^{J}{\mathcal{l}}_{i}\left(t\right)\right]}_{i=1}^{\mathcal{n}}$and similarly $\:{\mathfrak{T}=\left[{\int\:}_{t=J}^{{T}_{e}}{\mathcal{l}}_{i}\left(t\right)\right]}_{i=1}^{\mathcal{n}}$, where $\:Q$, $\:J$, and $\:{T}_{e}$ are as introduced before, $\:\mathcal{n}$ is the dimension of the given lead system $\:{\mathcal{L}}_{\dots\:\:}$, and $\:{\mathcal{l}}_{i}$ it its $\:i$-th lead. The QRS-T angle was then calculated as $\:\sphericalangle\:(\mathfrak{Q},\mathfrak{T})$.

Secondly, we considered an $\:\mathcal{n}$-dimensional vector $\:{\left[{\mathcal{l}}_{i}\left(t\right)\right]}_{i=1}^{\mathcal{n}}$for each sample $\:t$ of the lead system $\:{\mathcal{L}}_{\dots\:\:}={\left\{{\mathcal{l}}_{\mathcal{i}}\right\}}_{i=1}^{\mathcal{n}}$, and computed the QRS-T angle by using the dual integral formula described in the section of the Concepts of QRS-T angle computation.

Combination of the four lead systems and these two approaches to the angle computation gave us 8 different angle values for each of the beats of the selected ECG segments. In the subsequent text, we shall denote these values by symbols Area_XYZ_, Area_SVD_, Area_SVD−8_, Area_8 − leads_, Integral_XYZ_, Integral_SVD_, Integral_SVD−8_, and Integral_8 − leads_.

Intentionally, we are not reporting here on the concept of selecting QRS and T wave vectors based on the maximal magnitudes of vectors $\:{\left[{\mathcal{l}}_{i}\left(t\right)\right]}_{i=1}^{\mathcal{n}}$ because of the earlier observations that this concept leads to significantly poorer stability and reproducibility compared to the Area_…_ and Integral_…_ methods^26^. The Area_XYZ_, Area_SVD_ methods, and likewise the Integral_XYZ_, Integral_SVD_, methods are the same as previously investigated^26^. We are using these methods here solely for the comparison with the Area_SVD−8_, Area_8−leads_, and Integral_SVD−8_, and Integral_8−leads_ methods.

### Heart rate dependency

The previous comparison of 3-dimensional methods for QRS-T angle computation was based on regression residuals when relating the angles to the underlying heart rate^26^.

For each analysed beat in all ECG segments described in the section on QRS and T wave measurements, a 5-min history of preceding RR intervals was obtained. To make the results consistent with the previous heart-rate dependency analyses^26,39–41^, two concepts of underlying heart rate were considered. Individual measurements of the QRS-T angles were related to (A) the averages of preceding RR interval calculated for the number of preceding intervals ranging from 1 to 280 (interval-based averaging window) or calculating the RR interval average over the preceding 1 to 280 s (time-based averaging window), and (B) to the RR interval duration derived from exponential hysteresis models^39–41^ ranging the hysteresis constants (i.e., the time intervals leading to 95% adaptation after an abrupt heart rate change) either from 1 to 280 RR intervals (interval-based hysteresis) or from 1 to 280 s (time-based hysteresis)^39,42^.

Consistent with the previous analysis^26^, we have related the values of the QRS-T angles to the underlying heart rate (represented by the described expressions of RR interval durations) using a subject-specific linear second-degree polynomial regression in the form $\:{{\Theta\:}}_{i}=\:{\beta\:}_{0}+{\beta\:}_{1}{\varrho\:}_{i}+{\beta\:}_{2}{\varrho\:}_{i}^{2}+{\epsilon\:}_{i}$, where $\:{{\Theta\:}}_{i}$ are individual measurements of the QRS-T angle in the same study participant, $\:{\varrho\:}_{i}$ are the RR interval expressions of the underlying heart rate corresponding to the $\:i$-th measurement of the angle, $\:{\beta\:}_{0}$, $\:{\beta\:}_{1}$, and $\:{\beta\:}_{2}$ are regression coefficients optimised for the given subject, and $\:{\epsilon\:}_{i}$ are zero-centred regression errors. The intra-subject regression residuals (in the form $\:{\sigma\:=\left[\sum\:_{i}\left({\epsilon\:}_{i}^{2}\right)\right]}^{1/2}\:$) were used to judge the intra-subject precision and reproducibility of the individual expressions of the QRS-T angle. To distinguish between intra-subject residuals of the QRS-T/RR regressions based on angle calculations utilising different leads systems, we shall use symbols $\:{\sigma\:}_{XYZ}$, $\:{\sigma\:}_{SVD}$, and so on. Where needed, superscripts are added to indicate the method of computation, e.g., $\:{\sigma\:}_{8-leads}^{Area}$ or $\:{\sigma\:}_{8-leads}^{Integral}$.

The hysteresis profiles do not differ from the instantaneous heart rate if it has been stable over the preceding period^42^. Therefore, the calculations of the regression residuals were calculated for both all QRS-T angle measurements as well as just for those that were preceded by heart rate changes.

Intra-subject regression to the RR interval representing the underlying heart rate also allowed to interpolate the value of the QRS-T angle at different heart rates. This was applied only to the results based on the $\:{\mathcal{L}}_{8-\mathrm{l}\mathrm{e}\mathrm{a}\mathrm{d}\mathrm{s}\:}$lead system. Individually optimal interval-based hysteresis model (i.e., setting that led, in each subject separately, to the lowest $\:{\sigma\:}_{8-leads}$) was used for this purpose.

### Short-term variability

Having the individual processed QRS-T beats derived from 10-s segments of the original long-term ECG recordings allowed us to study the short-term (i.e., 10-s) variability of QRS-T angles. We used standard deviation (SD) of the individual QRS-T angle measurements. To study the dependency of these value on the underlying RR interval variability, we also calculated SD of individual RR intervals in the corresponding 10-s ECG segments (we shall use the SDNN acronym^43^ for this RR variability since, as already described, all the processed ECG segments contained only normal-to-normal RR intervals between beats of sinus rhythm origin).

In each study subject, the SD values of different QRS-T angle expressions were also averaged and related to the individually averaged SDNN values. This allowed us to investigate whether the short-term RR interval variability was driving the QRS-T angle variability.

### Statistics and data presentation

For the computation of the different versions of the QRS-T angles as well as of the hysteresis-based RR interval values, a purpose-specific software was developed in C++. Microsoft visual studio (version 17.4.3) with Visual C + + 2022 compiler (version 00476-80000-00000-AA606) was used. All calculations were made in double precision, the QRS-T angles were expressed in degrees (ranging from 0^o^ to 180^o^) and their numerical values were calculated with the precision of 8 decimal digits.

Descriptive data are presented as means ± standard deviation (SD); graphical images show means ± standard error of mean (SEM). Comparisons between different the results of different QRS-T angle calculations were graphically displayed using Bland–Altman type of presentation^44^. Intra-subject comparisons, e.g., the comparisons between regressions errors of the QRS-T/RR models for different QRS-T angle calculations, were evaluated using paired two-tail *t*-tests. Comparison between female and male sub-populations were assessed by two-sample two-tail *t*-tests assuming different variances of the compared samples. Where appropriate, Pearson correlation coefficients were used to study relationship between separate measurements.

Statistical tests used IBM SPSS package, version 29.0. P-values below 0.05 were considered statistically significant. Because of interdependence between the different indices, no correction for multiplicity of statistical testing was made.

## Results

### Population and electrocardiographic data

As stated, the source studies investigated 523 healthy volunteers including 259 females. There were no statistical age differences between females (33.4 ± 9.1 years) and males (33.7 ± 7.8 years).

The study was based on the total of 659,313 individual 10-second ECG samples and the total of 7,350,733 individual beats accepted for the analysis. On average, there were 14,298 ± 2983 and 13,825 ± 2981 individual beats processed per each female and male subject, respectively (no statistical differences between the sexes).

### Comparison of QRS-T angle expressions

No differences were found between the QRS-T angle values calculated based on the $\:{\mathcal{L}}_{\mathrm{S}\mathrm{V}\mathrm{D}-8\:}$and $\:{\mathcal{L}}_{8-\mathrm{l}\mathrm{e}\mathrm{a}\mathrm{d}\mathrm{s}\:}$lead systems. In more detail, with the 8 decimal digit precision, we have not found any instances in which the Area_SVD−8_ and Area_8 − leads_ would show any numerical differences. In 30 individual QRS-T beats (all in different study subjects), that is in fewer than 0.0005% of the processed ECG beats, we found a difference of ± 0.000001° between Integral_SVD−8_ and Integral_8 − leads_. We attributed these differences to the numerical precision of the calculation. In all other instances, the values of Integral_SVD−8_ and Integral_8 − leads_ were identical.

**Fig. 1:**
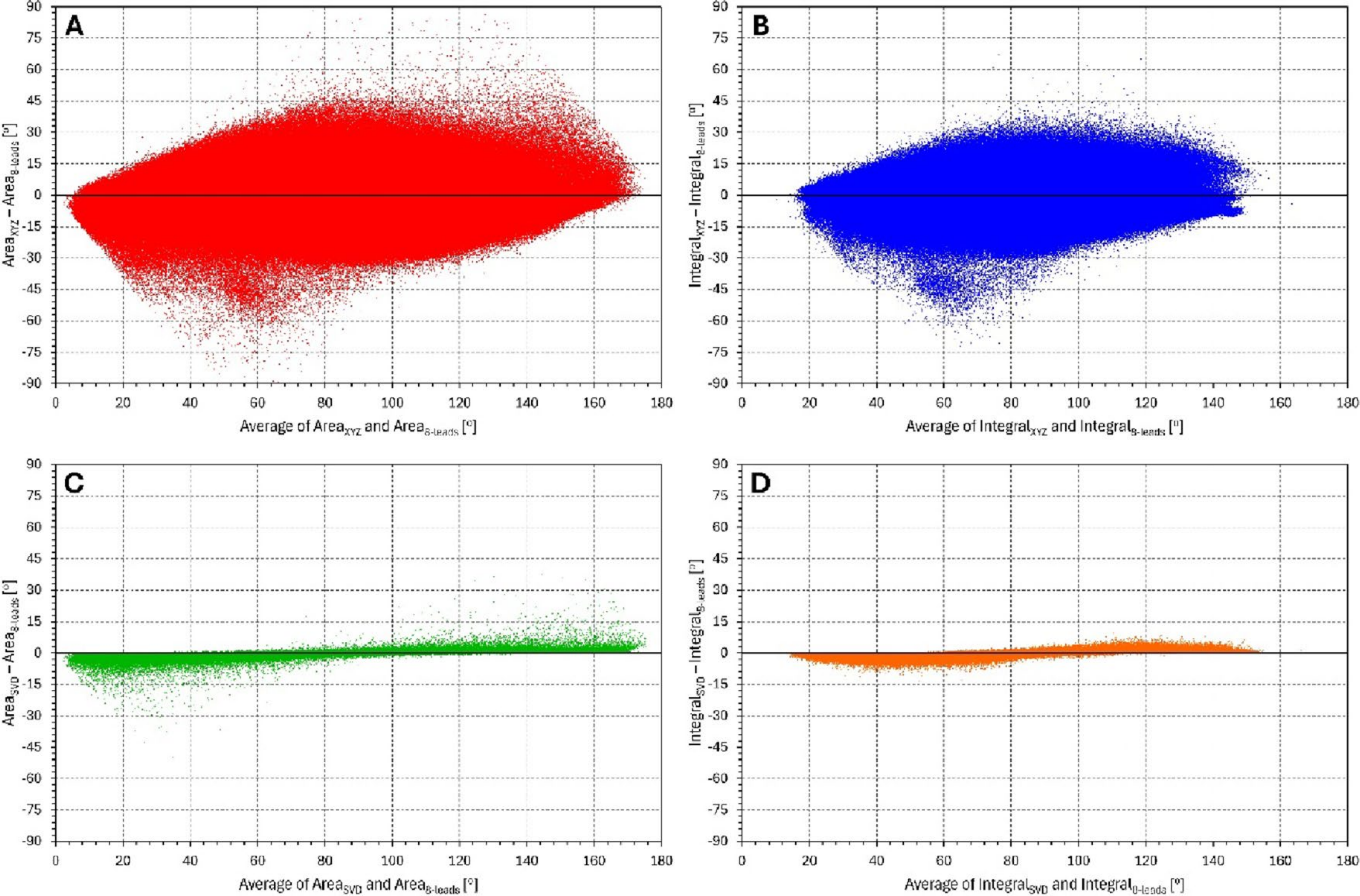
Bland-Altman type of scatter diagrams showing comparisons between individual results of QRS-T angle measurements in individual beats processed in the study. Panels (**A**–**D**) show the comparisons of Area_XYZ_ and Area_8 − leads_, Integral_XYZ_ and Integral_8 − leads_, Area_SVD_ and Area_8 − leads_, and Integral_SVD_ and Integral_8 − leads_, respectively.

Figure 1 shows the comparisons between the $\:{\mathcal{L}}_{8-\mathrm{l}\mathrm{e}\mathrm{a}\mathrm{d}\mathrm{s}\:}$derived QRS-T angles and the corresponding angle expressions based on $\:{\mathcal{L}}_{\mathrm{X}\mathrm{Y}\mathrm{Z}\:}$and $\:{\mathcal{L}}_{\mathrm{S}\mathrm{V}\mathrm{D}}$. The figure shows comparisons when the results of all investigated beats were pooled together. Because of the known heart rate dependency of the QRS-T angles and because the individual beats differed in the underlying heart rate, statistical evaluation of the differences shown in the Figure are of limited informative value. The Figure mainly shows that the $\:{\mathcal{L}}_{8-\mathrm{l}\mathrm{e}\mathrm{a}\mathrm{d}\mathrm{s}\:}$based results are substantially closer to those based on $\:{\mathcal{L}}_{\mathrm{S}\mathrm{V}\mathrm{D}\:}$rather than on $\:{\mathcal{L}}_{\mathrm{X}\mathrm{Y}\mathrm{Z}}$. Indeed, the averaged differences between the expressions based on $\:{\mathcal{L}}_{\mathrm{X}\mathrm{Y}\mathrm{Z}\:}$and $\:{\mathcal{L}}_{8-\mathrm{l}\mathrm{e}\mathrm{a}\mathrm{d}\mathrm{s}\:}$were − 1.33 ± 8.89° and − 1.40 ± 7.18° for the Area_…_ and Integral_…_ methods, respectively, which was close to the differences between the expressions based on $\:{\mathcal{L}}_{\mathrm{X}\mathrm{Y}\mathrm{Z}\:}$and $\:{\mathcal{L}}_{\mathrm{S}\mathrm{V}\mathrm{D}\:}$(− 1.26 ± 8.89° and − 1.05 ± 7.21°). The averaged differences between the expressions based on $\:{\mathcal{L}}_{\mathrm{S}\mathrm{V}\mathrm{D}\:}$and $\:{\mathcal{L}}_{8-\mathrm{l}\mathrm{e}\mathrm{a}\mathrm{d}\mathrm{s}\:}$were − 0.07 ± 0.34° and − 0.34 ± 0.52°, again for the Area_…_ and Integral_…_ methods, respectively. Considering the identity of $\:{\mathcal{L}}_{8-\mathrm{l}\mathrm{e}\mathrm{a}\mathrm{d}\mathrm{s}\:}$and $\:{\mathcal{L}}_{\mathrm{S}\mathrm{V}\mathrm{D}-8\:}$results, the closeness of the expressions based on $\:{\mathcal{L}}_{\mathrm{S}\mathrm{V}\mathrm{D}\:}$and $\:{\mathcal{L}}_{8-\mathrm{l}\mathrm{e}\mathrm{a}\mathrm{d}\mathrm{s}\:}$ is not surprising since the 4-th till 8-th components of the SVD decomposition represent, by definition, smaller proportions of the original ECG signals.

Since the QRS-T angle results based on $\:{\mathcal{L}}_{8-\mathrm{l}\mathrm{e}\mathrm{a}\mathrm{d}\mathrm{s}\:}$and $\:{\mathcal{L}}_{\mathrm{S}\mathrm{V}\mathrm{D}\:}$were close together, the physiology observations reported previously for the $\:{\mathcal{L}}_{\mathrm{S}\mathrm{V}\mathrm{D}\:}$results^26,45,46^ apply also to the angle values based on $\:{\mathcal{L}}_{8-\mathrm{l}\mathrm{e}\mathrm{a}\mathrm{d}\mathrm{s}}$. Therefore, we shall further concentrate on the precision of the angle estimates as quantified by the QRS-T/RR angle regression residuals.

### QRS-T/RR angle regression residuals (RR interval averages)

**Fig. 2:**
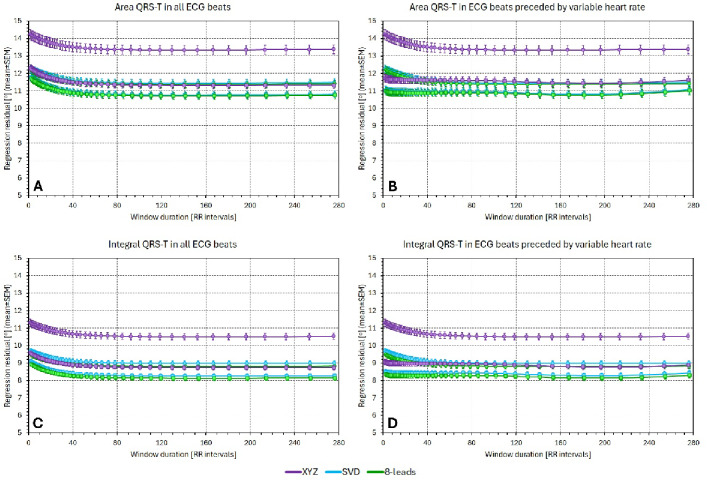
The figure shows regression residuals for different QRS-T angle expressions related to differently long averages of preceding RR intervals. The horizontal axes of the panels show the number of averaged RR intervals; the vertical axes show the regression residuals. Panels (**A**) and (**C**) show residuals of regressions involving all individual processed beats, panels (**B**) and (**D**) residuals of regression calculated over beats preceded by variable heart rates. Panels (**A**) and (**B**) show the residuals of the Area_…_ methods, panels (**C**) and (**D**) of the Integral_…_ methods. In all panels, the violet, cyan, and green graphs show the $\:{\sigma\:}_{\mathrm{X}\mathrm{Y}\mathrm{Z}}$, $\:{\sigma\:}_{\mathrm{S}\mathrm{V}\mathrm{D}}$, and $\:{\sigma\:}_{8-\mathrm{l}\mathrm{e}\mathrm{a}\mathrm{d}\mathrm{s}}$ residuals, respectively. The graphs with circles and squares show the results for the female and male study subpopulation, respectively.

Figure 2 shows regression residuals when relating the QRS-T angle values to averages of preceding RR intervals. The figure compares the $\:{\mathcal{L}}_{8-\mathrm{l}\mathrm{e}\mathrm{a}\mathrm{d}\mathrm{s}\:}$results with those based on $\:{\mathcal{L}}_{\mathrm{X}\mathrm{Y}\mathrm{Z}}$ and $\:{\mathcal{L}}_{\mathrm{S}\mathrm{V}\mathrm{D}}$. That is, the figure shows population summaries (mean ± SEM) of $\:{\sigma\:}_{\mathrm{X}\mathrm{Y}\mathrm{Z}},$ $\:{\sigma\:}_{\mathrm{S}\mathrm{V}\mathrm{D}}$, and $\:{\sigma\:}_{8-\mathrm{l}\mathrm{e}\mathrm{a}\mathrm{d}\mathrm{s}}$. (The figure presents the results based on the calculation of RR interval averages over a given number of preceding RR intervals. The results based on RR interval averages over a given time period are not shown since they closely replicated the results presented in Fig. 2.)

Figure 2 shows that in both females and males, the residuals of the QRS-T/RR regressions based on $\:{\mathcal{L}}_{8-\mathrm{l}\mathrm{e}\mathrm{a}\mathrm{d}\mathrm{s}\:}$were lower compared to those based on the other lead systems, regardless of whether the calculations involved all ECG beats or only those beats that were preceded by variable heart rate.

**Fig. 3:**
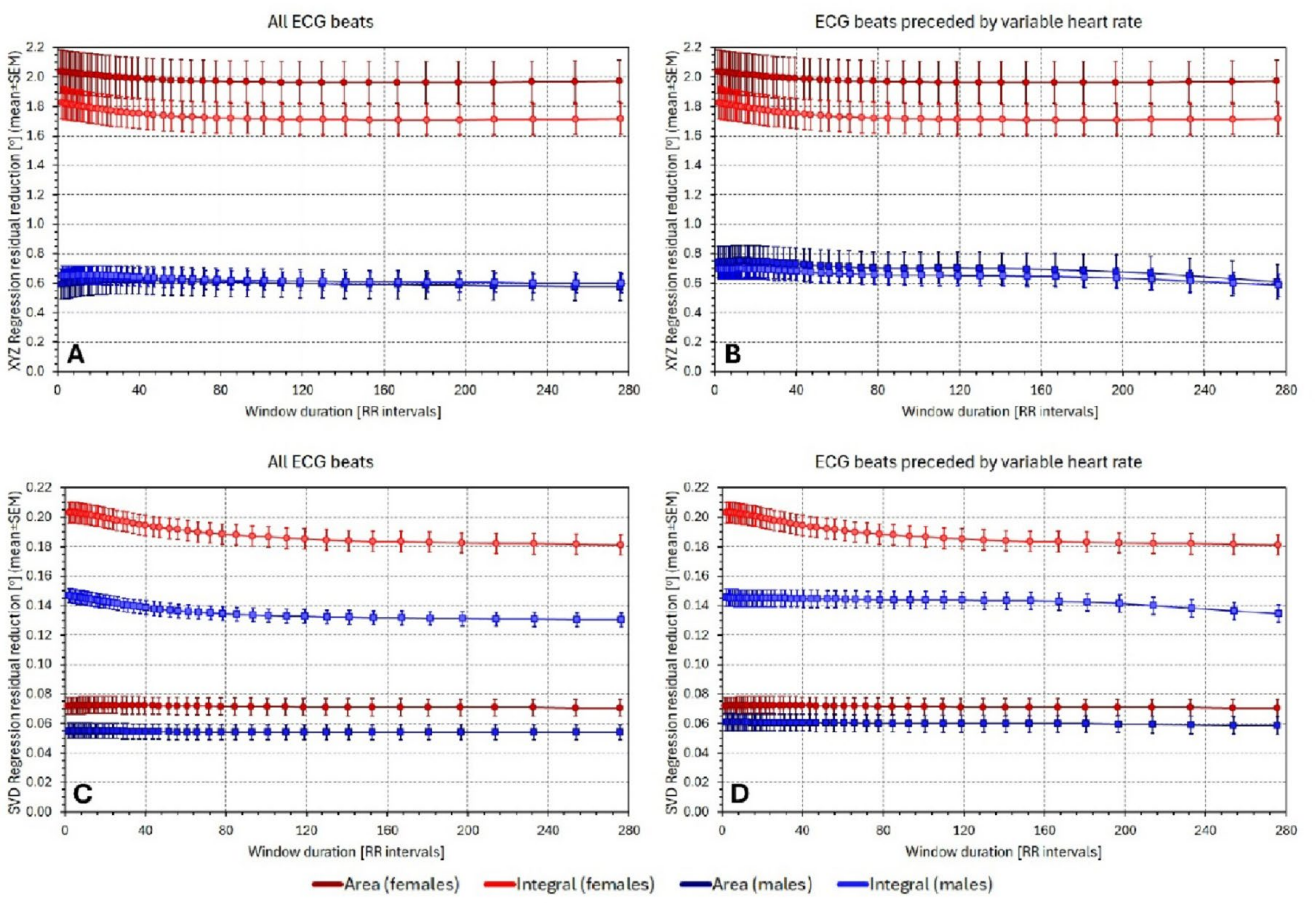
The figure shows the differences in regression residuals shown in Fig. 2, that is, the differences $\:{\sigma\:}_{\mathrm{X}\mathrm{Y}\mathrm{Z}}-{\sigma\:}_{8-\mathrm{l}\mathrm{e}\mathrm{a}\mathrm{d}\mathrm{s}}$ in panels (**A**) and (**B**), and $\:{\sigma\:}_{\mathrm{S}\mathrm{V}\mathrm{D}}-{\sigma\:}_{8-\mathrm{l}\mathrm{e}\mathrm{a}\mathrm{d}\mathrm{s}}$ in panels (**C**) and (**D**). Panels (**A**) and (**C**) show the residuals of regressions involving all individual processed beats, panels (**B**) and (**D**) residuals of regression calculated over beats preceded by variable heart rates (compare with the layout of Fig. 2). Dark red and blue colours show the $\:{\sigma\:}_{\dots\:}^{Area}-{\sigma\:}_{8-\mathrm{l}\mathrm{e}\mathrm{a}\mathrm{d}\mathrm{s}}$ values, the light red and blue colours show the $\:{\sigma\:}_{\dots\:}^{Integral}-{\sigma\:}_{8-\mathrm{l}\mathrm{e}\mathrm{a}\mathrm{d}\mathrm{s}}$ values. The red graphs with circles and blue graphs with squares show the results for the female and male study subpopulation, respectively.

The corresponding comparisons of the residual differences are shown in Fig. 3. This Figure shows the statistical population summaries (mean ± SEM) of intra-subject differences $\:{\sigma\:}_{\mathrm{X}\mathrm{Y}\mathrm{Z}}-$ $\:{\sigma\:}_{8-\mathrm{l}\mathrm{e}\mathrm{a}\mathrm{d}\mathrm{s}}$ and similarly $\:{\sigma\:}_{\mathrm{S}\mathrm{V}\mathrm{D}}-$ $\:{\sigma\:}_{8-\mathrm{l}\mathrm{e}\mathrm{a}\mathrm{d}\mathrm{s}}$. It can be seen in the Figure that all lower SEM bands were clearly above zero. Indeed, all corresponding statistical tests showed highly significant reductions of $\:{\sigma\:}_{8-\mathrm{l}\mathrm{e}\mathrm{a}\mathrm{d}\mathrm{s}}$ compared to $\:{\sigma\:}_{\mathrm{X}\mathrm{Y}\mathrm{Z}}$ and to $\:{\sigma\:}_{\mathrm{S}\mathrm{V}\mathrm{D}}$ (*p* < 0.0001 for all individual comparisons).

### QRS-T/RR angle regression residuals (hysteresis corrected RR intervals)

**Fig. 4:**
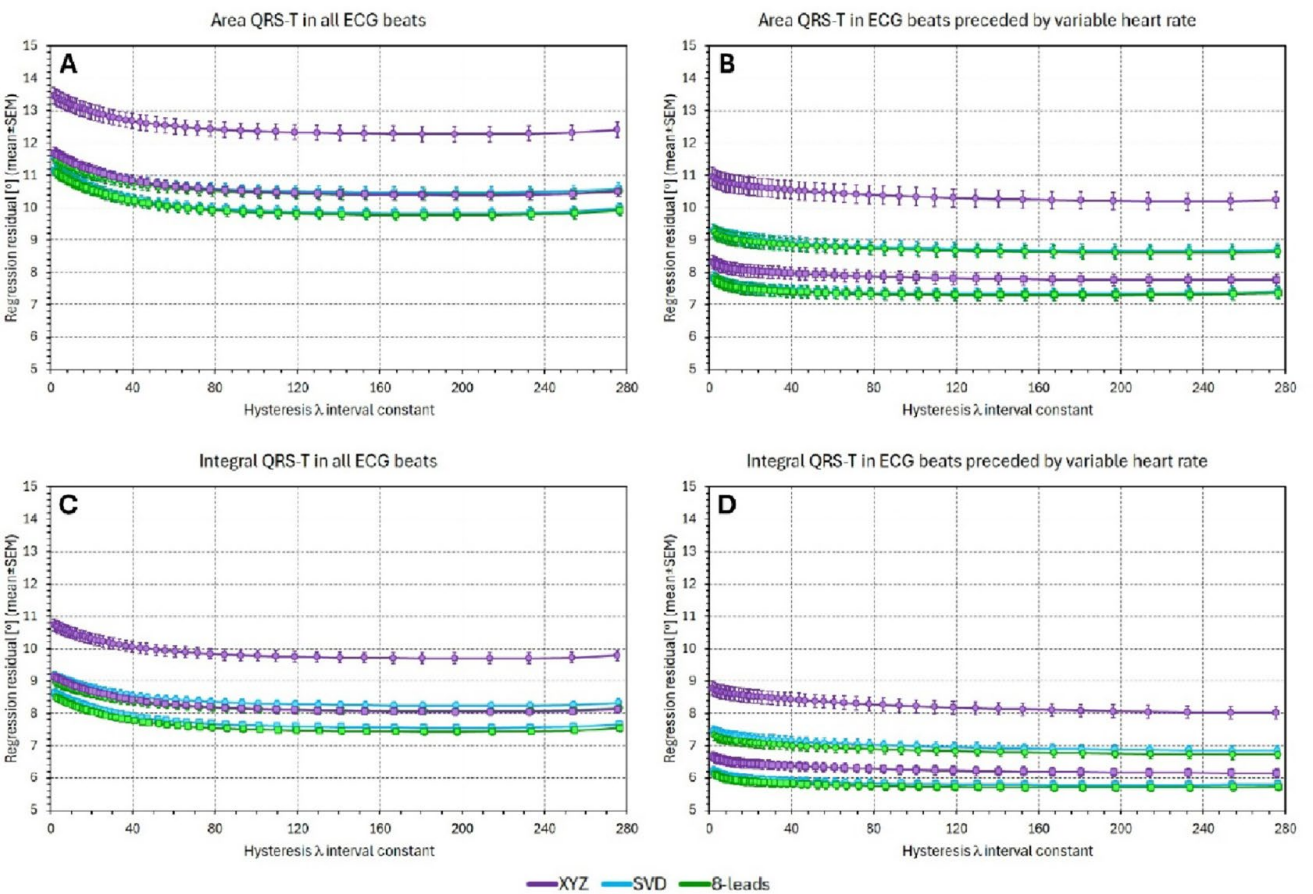
The figure shows regression residuals for different QRS-T angle expressions related to different interval-based hysteresis constants of preceding RR intervals. The horizontal axes of the panels show the number of the RR intervals used in the exponential decay hysteresis model; the vertical axes show the regression residuals. Panels (**A**) and (**C**) show residuals of regressions involving all individual processed beats, panels (**B**) and (**D**) residuals of regression calculated over beats preceded by variable heart rates. Panels (**A**) and (**B**) show the residuals of the Area_…_ methods, panels (**C**) and (**D**) of the Integral_…_ methods. In all panels, the violet, cyan, and green graphs show the $\:{\sigma\:}_{\mathrm{X}\mathrm{Y}\mathrm{Z}}$, $\:{\sigma\:}_{\mathrm{S}\mathrm{V}\mathrm{D}}$, and $\:{\sigma\:}_{8-\mathrm{l}\mathrm{e}\mathrm{a}\mathrm{d}\mathrm{s}}$ residuals, respectively. The graphs with circles and squares show the results for the female and male study subpopulation, respectively. Compare with Fig. 2.

**Fig. 5:**
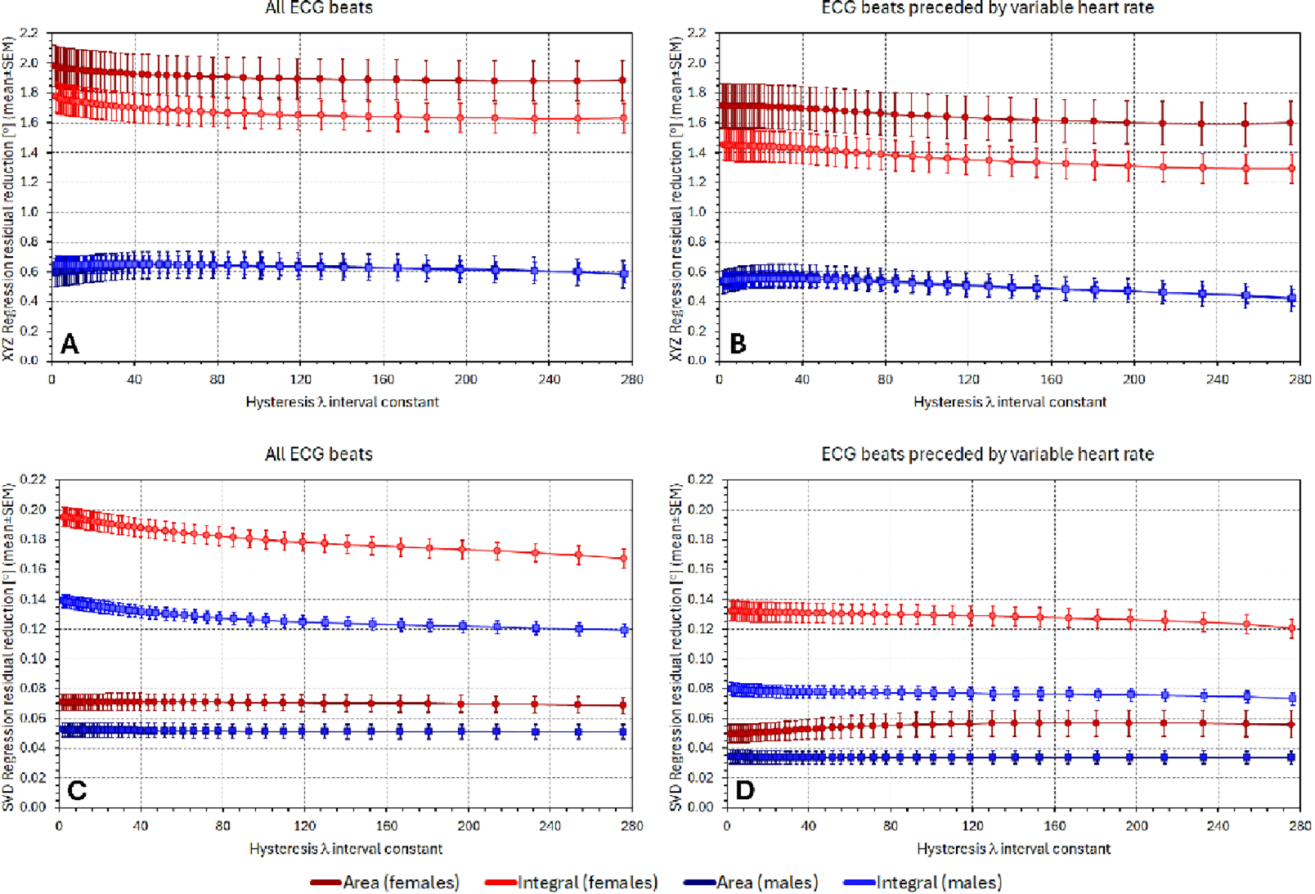
The figure shows the differences in regression residuals shown in Fig. 4, that is, the differences $\:{\sigma\:}_{\mathrm{X}\mathrm{Y}\mathrm{Z}}-{\sigma\:}_{8-\mathrm{l}\mathrm{e}\mathrm{a}\mathrm{d}\mathrm{s}}$ in panels (**A**) and (**B**), and $\:{\sigma\:}_{\mathrm{S}\mathrm{V}\mathrm{D}}-{\sigma\:}_{8-\mathrm{l}\mathrm{e}\mathrm{a}\mathrm{d}\mathrm{s}}$ in panels (**C**) and (**D**). Panels (**A**) and (**C**) show the residuals of regressions involving all individual processed beats, panels (**B**) and (**D**) residuals of regression calculated over beats preceded by variable heart rates (compare with the layout of Fig. 4). Dark red and blue colours show the $\:{\sigma\:}_{\dots\:}^{Area}-{\sigma\:}_{8-\mathrm{l}\mathrm{e}\mathrm{a}\mathrm{d}\mathrm{s}}$ values, the light red and blue colours show the $\:{\sigma\:}_{\dots\:}^{Integral}-{\sigma\:}_{8-\mathrm{l}\mathrm{e}\mathrm{a}\mathrm{d}\mathrm{s}}$ values. The red graphs with circles and blue graphs with squares show the results for the female and male study subpopulation, respectively. Compare with Fig. 3.

Figures 4 and 5 are the counterparts of Figs. 2 and 3. Instead of RR interval averages over a given number of preceding RR intervals, Figs. 4 and 5 use RR interval durations derived from interval-based RR hysteresis profiles. (I.e., instead of simple averages of RR intervals, weighted averages are used using the weights derived from exponential decay models.) Comparisons of Fig. 4 with Fig. 2, and similarly of Fig. 5 with Fig. 3 show that (a) the hysteresis-based RR interval estimates lead to lower regression residuals compared to simple RR interval averages, and (b) the results comparing the $\:{\sigma\:}_{\mathrm{X}\mathrm{Y}\mathrm{Z}},$ $\:{\sigma\:}_{\mathrm{S}\mathrm{V}\mathrm{D}}$, and $\:{\sigma\:}_{8-\mathrm{l}\mathrm{e}\mathrm{a}\mathrm{d}\mathrm{s}}$ values are in principle the same. At each hysteresis setting, the $\:{\sigma\:}_{8-\mathrm{l}\mathrm{e}\mathrm{a}\mathrm{d}\mathrm{s}}$ values are, in intra-subject paired comparisons, significantly smaller than the $\:{\sigma\:}_{\mathrm{X}\mathrm{Y}\mathrm{Z}}\:$and $\:{\sigma\:}_{\mathrm{S}\mathrm{V}\mathrm{D}}$ values. In all individual comparisons, p-value < 0.0001 was again obtained. (The Figs. 4 and 5 show the results based on interval-based hysteresis. Results based on time-based hysteresis were practically identical and are therefore not shown.)

### Differences between Area_8-leads_ and Integral_8-leads_

**Fig. 6:**
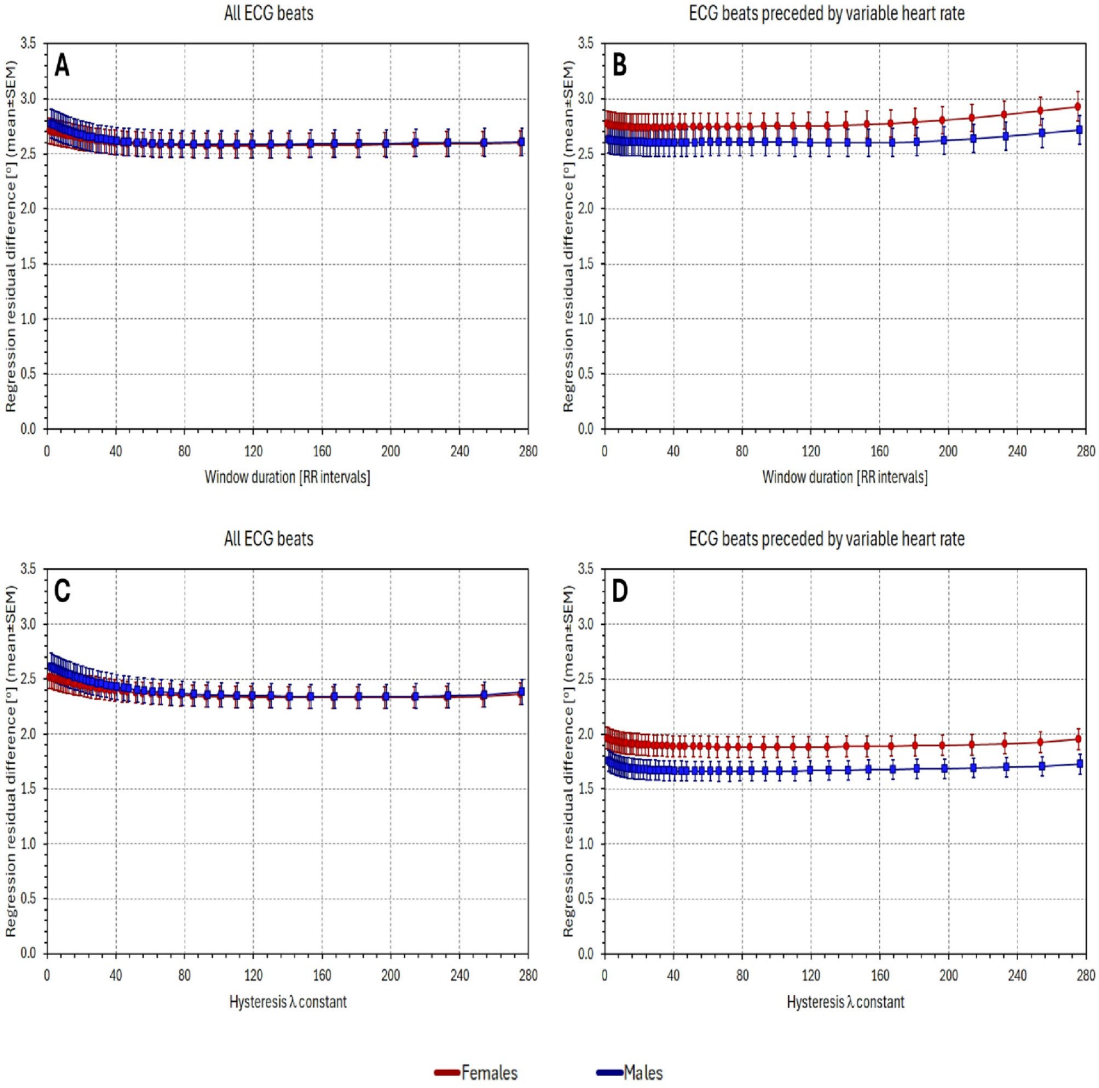
The figure summarises the differences $\:{\sigma\:}_{8-leads}^{Area}-{\sigma\:}_{8-leads}^{Integral}$. The values shown in panels (**A**–**D**) correspond to those shown in panels A and C of Fig. 2, in panels B and D of Fig. 2, panels A and C of Fig. 4, and panels B and D of Fig. 4, respectively. In all panels of the figure, red graphs with circles and blue graphs with squares show the results for the female and male study subpopulation, respectively.

Figure 6 shows the differences between $\:{\sigma\:}_{8-leads}^{Area}$ and $\:{\sigma\:}_{8-leads}^{Integral}$. Consistently, the Integral_8 − leads_ method led to lower regression residua compared to the Area_8 − leads_ method. As easily seen in the Figure, all the statistical comparisons were highly statistically significant (*p* < 0.0001 for all individual comparisons).

**Fig. 7:**
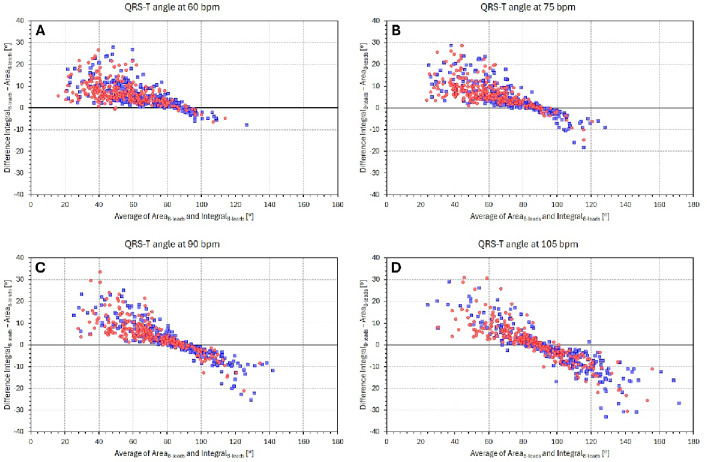
Bland–Altman type of scatter diagrams between subject-specific regression-based projections of Area_8 − leads_ and Integral_8 − leads_ values to 60 bpm (**A**), 75 bpm (**B**), 90 bpm (**C**), and 105 bpm (**D**). Red circles and blue squares correspond to individual female and male study subjects, respectively.

Figure 7 presents Bland–Altman type of scatter diagrams between the Area_8 − leads_ and Integral_8 − leads_ methods using data of individual interpolations of the QRS-T/RR regressions to different levels of underlying heart rates. When increasing the heart rate to which the individual interpolations were made, an obvious shift of the bias between the two computation methods is visible.

### 10-second variability

**Fig. 8:**
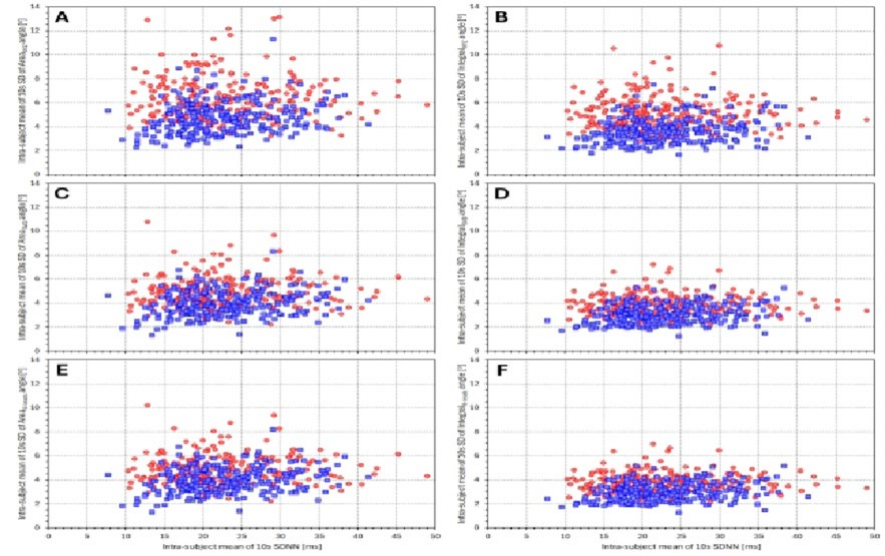
Scatter diagrams between subject-specific averages of 10-s SDNN and of 10-s SD of different QRS-T angle expressions. The horizontal axes of all panels correspond to the 10-s SDNN values, the vertical axes of panels (**A**–**F**) correspond to the averaged 10-s SDs of Area_XYZ_, Integral_XYZ_, Area_SVD_, Integral_SVD_, Area_8 − leads_, and Integral_8 − leads_, respectively. In all panels, the read circles and blue squares correspond to female and male study subjects, respectively.

Figure 8 shows the scatter diagrams between individual averages of 10-s SDNN and corresponding averages of 10-s SD of individual QRS-T angle expressions. When correlating the displayed values, we observed somewhat surprising sex differences. In females, the correlations between individual SDNN averages and QRS-T angle averages were − 0.035, − 0.063, 0.044, − 0.015, 0.045, and − 0.011 for Area_XYZ_, Integral_XYZ_, Area_SVD_, Integral_SVD_, Area_8 − leads_, and Integral_8 − leads_, respectively (all not significant). In males, however, the corresponding correlations were 0.231, 0.212, 0.239, 0.232, 0.242, and 0.234 (all *p* < 0.001).

**Fig. 9:**
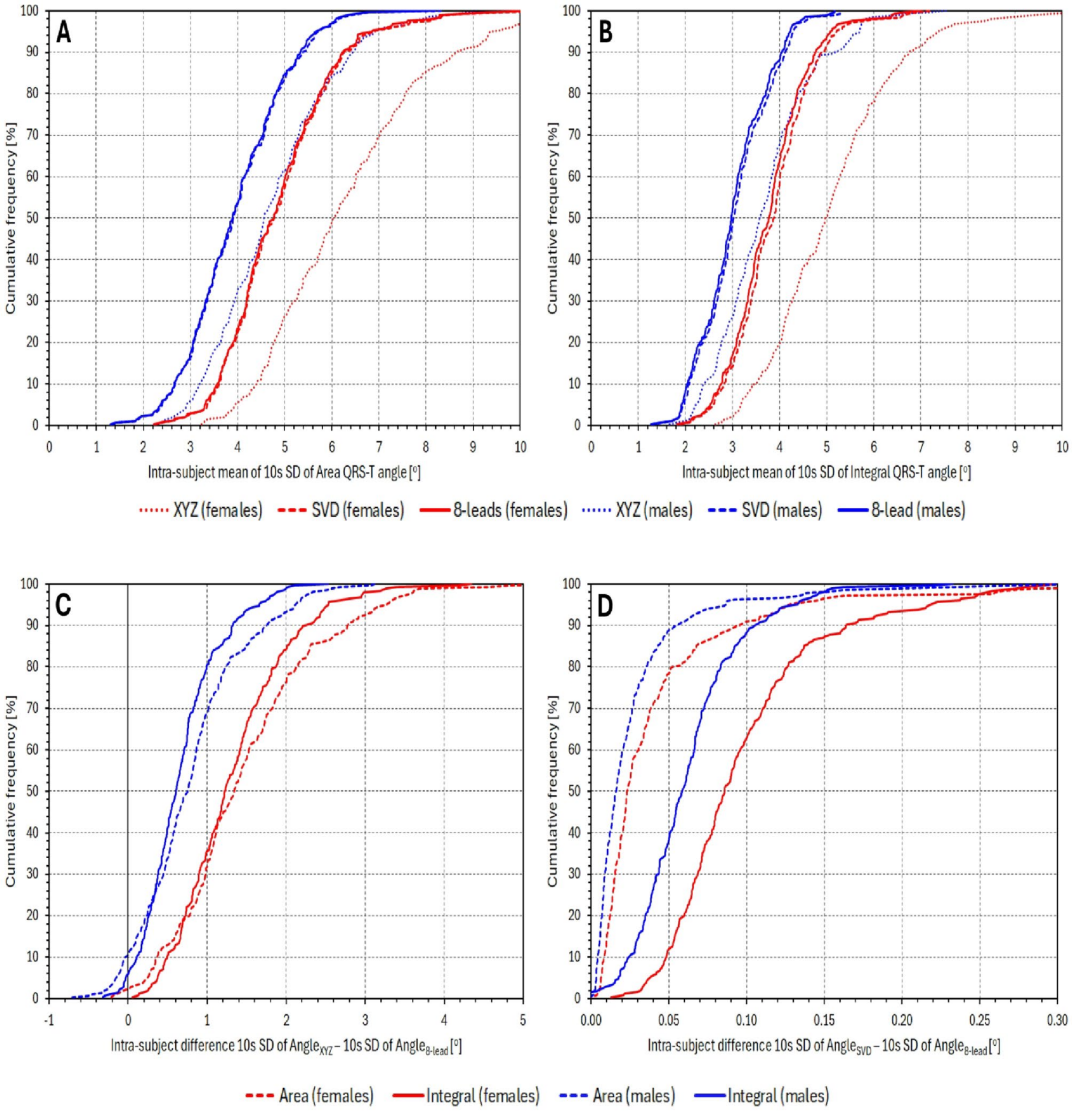
Cumulative population distributions of the intra-subject means of 10-s SDs of different QRS-T angle expressions. Panels (**A**) and (**B**) show these distributions for Area_…_ and Integral_…_ expressions, respectively. In these panels, dotted, dashed, and full lines correspond to …_XYZ_, …_SVD_, and …_8−leads_ versions, respectively. Panel (**C**) shows the differences …_XYZ_ – …_8−leads_ angle expressions, and panel (**D**) the differences …_SVD_ – …_8−leads_ angle expressions. In panels (**C**) and (**D**), the dashed and full lines correspond to Area_…_ and Integral_…_ expressions. In all panels, the red and blue lines correspond to female and male subpopulation, respectively.

Figure 9 shows the cumulative distributions of the intra-subject averages of the 10-second SD of individual QRS-T angle expressions. For Area_XYZ_, Integral_XYZ_, Area_SVD_, Integral_SVD_, Area_8 − leads_, and Integral_8 − leads_, the values of the intra-subject averages of the 10-s SD in females were 6.36 ± 1.85°, 5.11 ± 1.36°, 4.92 ± 1.22°, 3.87 ± 0.87°, 4.87 ± 1.19°, and 3.77 ± 0.83°, respectively. The corresponding values in males were 4.75 ± 1.32°, 3.69 ± 1.02°, 3.98 ± 1.06°, 3.08 ± 0.75°, 3.95 ± 1.05°, and 3.02 ± 0.73°, respectively. All the comparisons shown in the bottom panels of Fig. 9 were highly statistically significant (*p* < 0.0001 for all). In both males and females, the individually averaged 10-second SD of Area_8 − leads_ were also significantly larger than those of Integral_8 − leads_ (*p* < 0.0001 in both sexes).

**Fig. 10:**
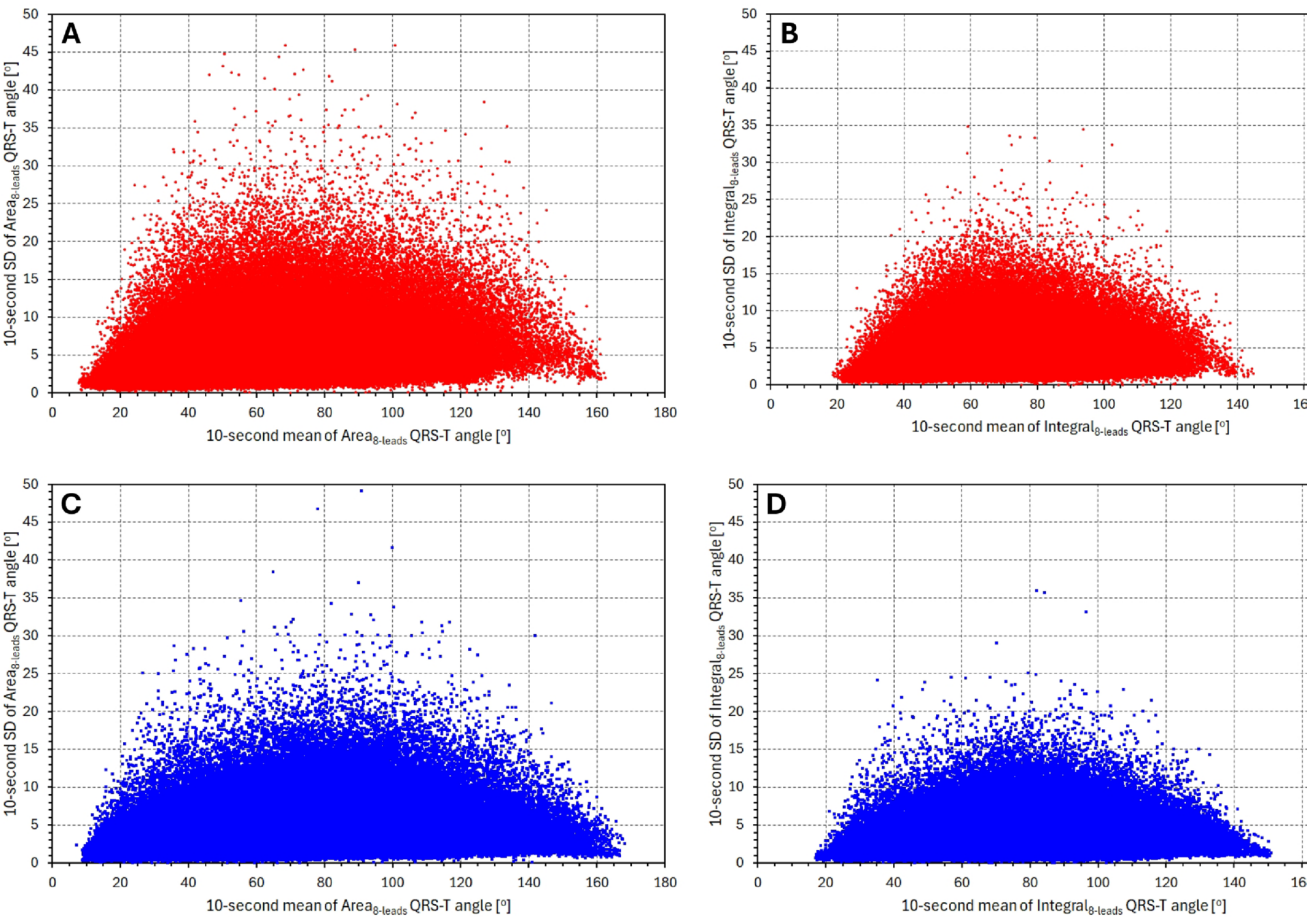
Panels (**A**) and (**C**) show the scatter diagrams between the 10-s means of Area_8 − leads_ and 10-s SD of Area_8 − leads_ in individual ECG segments processed in the study. The corresponding scatter diagrams for Integral_8 − leads_ are shown in panels (**B**) and (**D**). Panels (**A**) and (**B**) show results in female subjects, panels (**C**) and (**D**) in male subjects.

The difference between 10-second SD of Area_8 − leads_ and of Integral_8 − leads_ is also visually obvious in Fig. 10 which shows scatter diagrams between the 10-s averages and 10-s SDs of the angle measurements.

### Sex comparisons

**Fig. 11:**
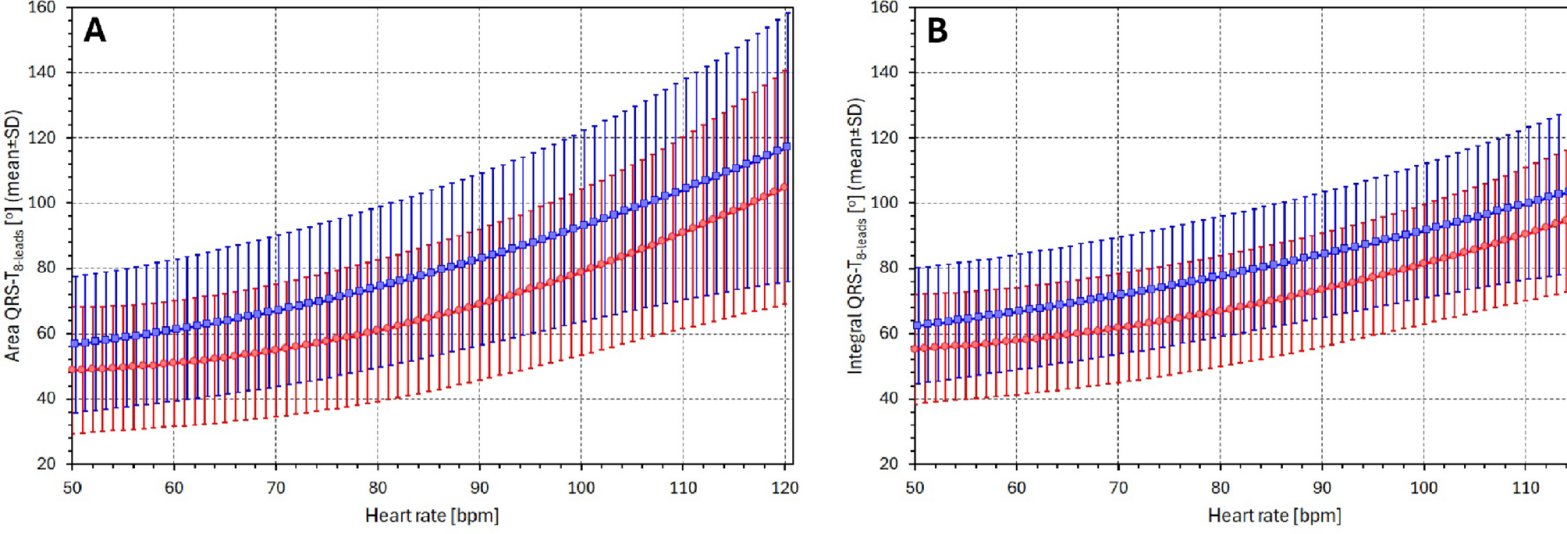
The Figure shows regression-based projections of Area_8 − leads_ (**A**) and Integral_8 − leads_ (**B**) to different levels of heart rate averaged for all female subjects (red curves) and all male subjects (blue curves).

Sex differences in QRS-T angle have been previously described^26,47,48^. To complement these reports, we repeated the comparison between females and males using individually interpolated Area_8 − leads_ and Integral_8 − leads_ angle values to heart rates ranging from 50 to 120 bpm. Results are shown in Fig. 11. At each of the interpolation heart rates, the sex differences were statistically significant (*p* < 0.001 for all comparisons).

## Discussion

The study shows that the QRS-T angle might be computed based on native 12-lead ECG signal (i.e., using only the algebraically independent leads) without the need for any transformation into orthogonal leads. Moreover, and equally importantly, the study also shows that the QRS-T angles computed in this way appear more stable with a lower intra-subject variability compared to the “usual” results based on orthogonal transformations. (Similar albeit numerically different results were also obtained with the angle computation based on the maximal vector magnitudes which we have not included in this report.) Consistent with the previous report on the different computational methods^26^, we have also shown that the Integral approach is more stable than the approach based on the areas under the ECG components.

The identity of the QRS-T angle calculations based on $\:{\mathcal{L}}_{\mathrm{S}\mathrm{V}\mathrm{D}-8\:}$and $\:{\mathcal{L}}_{8-\mathrm{l}\mathrm{e}\mathrm{a}\mathrm{d}\mathrm{s}\:}$validates our software implementation but is, in principle, not surprising. Each lead of $\:{\mathcal{L}}_{\mathrm{S}\mathrm{V}\mathrm{D}-8\:}$is a linear combination of the original native leads of $\:{\mathcal{L}}_{8-\mathrm{l}\mathrm{e}\mathrm{a}\mathrm{d}\mathrm{s}\:}$^20,21,38^. Such linear combinations should not, and indeed do not, have any effect on the multi-dimensional angles.

While this study used the ECGs obtained with Mason-Likar electrode configuration, the same signal processing principle is also applicable to 12-lead ECGs based on the standard electrode positions. When we used such data, the same essential comparisons were observed (data not presented). Technically, the same principle of combining different projections of the QRS complex and of the T wave into multidimensional vectors may also be applied to non-standard electrode positions including those used in different wearable ECG devices. It seems logical to conjecture that if such non-standard electrode configurations contain projections well outside a single plane, reasonable estimates of the spatial QRS-T angles might be obtained.

The concept of computing the spatial angle in a hyper-space of 8 dimensions is naturally beyond any possibility of mental visualization. Nevertheless, it is now apparent that considering all ECG leads together rather than one by one increases the potential medical applicability and allows deriving clinical observations that are beyond the “standard” ECG interpretation^38,49,50^. The reconstruction of 3 orthogonal leads, regardless of whether by a general transformation matrix or by signal-specific SVD, unavoidably loses some information contained in the native signals. Even when this information loss is minute, it is not without meaning^49^. We believe that the inclusion of all the ECG signals is the reason why the direct computation of the QRS-T angle using the original $\:{\mathcal{L}}_{8-\mathrm{l}\mathrm{e}\mathrm{a}\mathrm{d}\mathrm{s}\:}$leads appears more precise compared to other possibilities.

The reasons for the higher precision of the Integral method compared to the Area method (and similarly to the Maximum method)^26,36^ are likely caused by the incorporation of morphological details of both the QRS complex and the T wave. By using the area under the ECG curve, any fragmentations of the signals are omitted. On the contrary, the Integral method incorporates angles between individual signal instances of both QRS and T wave and provides, in essence, their weighted averaging. The simplicity of using only weights by the product of the vector magnitudes related to each integrated angle might deserve further attention. Although it guarantees that ECG signal aberrations in the low amplitude parts of the QRS complex and of the T wave are given lower weights compared to the dominant parts of the ECG components, further optimisation of the signal weights is clearly possible if not desirable.

The Integral method appears not only more precise compared to the Area method but also provides somewhat different results, as shown in Fig. 7. The trend to different bias levels between the two methods observed at different heart rates likely reflects the different heart rate dependent morphologies of the ECG components. The incorporation of the morphological nuances is also the likely reason for the lower short-term beat-to-beat variability of the computed angles. Figures 8 and 9 document not only the increased precision (in terms of beat-to-beat reproducibility) of the direct angle computation compared to the 3-dimensional conversions but also the increased precision of the Integral method compared to the Area method. Accepting the conclusion that the QRS-T angle computation based on the native leads of $\:{\mathcal{L}}_{8-\mathrm{l}\mathrm{e}\mathrm{a}\mathrm{d}\mathrm{s}\:}$increases the precision of the results, the graphs in Fig. 4 (and the almost identical results based on time-based QRS-T/RR hysteresis which we have not presented) support the previous conjecture that the QRS-T angle is not primarily dependent on heart rate but that it is influenced by the same autonomic and other regulatory mechanisms that lead to heart rate changes^26^.

Despite the statistical significance, the comparisons of the regression residuals might appear to be based on numerically small numbers. Nevertheless, these numbers are not those of individual ranges of the angles but the width of the curvilinear regression patterns at individual heart rate instances.

Both the Area and the Integral methods assume the same span of QRS complex and of the T wave in all ECG leads used in the computation. This negates the concept of the so-called QT dispersion which is now well understood to originate from isoelectric projections of the ECG signals in some leads as well as from measurement inaccuracies^51–54^.

The sex differences in the QRS-T angle are well known but their physiologic background is poorly understood. It is most likely somewhat different from the hormonal reasons for the known sex difference in the rate corrected QTc interval^55,56^ since the pattern of heart rate dependency of sex-differences of the QTc interval^55^ appears different to the patterns that we have presented in Fig. 11. Further studies are needed to understand these differences as well as many other physiologic properties of the QRS-T angle including age-dependency, development in children, hormonal changes (e.g., during menstrual cycle or sex transition procedures), electrolyte influences, and drug effects.

### Limitations

Limitations of the present study also need to be considered. The main study limitation is the analysis of data obtained from healthy volunteers. While this allowed technical and physiologic factors to be incorporated into the study, we are unable to comment on the risk-prediction differences between the different modes of calculation. It can only be speculated that measurements that appear more stable and reproducible in a physiologic study of healthy subjects will prove to be similarly superior in clinical applications. While we distinguished sex of the study subjects in individual analyses, we have not considered the relationship of the results to the age of the subjects^57^, their race^58,59^, and body composition^60^. As already stated, all these factors deserve to be investigated in subsequent physiological studies. The Integral method would also allow to study the angle differences between different sections of the ECG components (e.g., the ascending and descending parts of the T wave). This was not considered in this investigation. To investigate the 10-s variability of QRS-T angles, we used only simple SD. Other possibilities, such as the root mean square of successive differences^43^ would also be possible.

## Conclusion

The study shows convincingly that no orthogonal transformation is needed when computing the QRS-T angle from standard 12-lead ECGs. Direct angle computation from the native ECG leads that are directly recorded and algebraically independent (i.e., leads I, II, V1, V2, …, V6) appears more stable in the relationship to heart rate and shows lower beat-to-beat variability when applied to individual QRS-T complexes.

## Data Availability

The source ECG data analysed in the study will be made available by the authors. Requests for the data are to be sent to the corresponding author.
